# Energy Drink Use and Adolescent Sleep Debt: Implications for Obesity, Cognition, and Physical Activity

**DOI:** 10.3390/nu18182946

**Published:** 2026-09-08

**Authors:** Giorgos K. Sakkas, Ilias Ntoumas, Christoforos D. Giannaki, Christina Karatzaferi

**Affiliations:** 1 Department of Physical Education and Sport Science, University of Thessaly, GR42100 Trikala, Thessaly, Greece; intoumas@uth.gr (I.N.); ck@uth.gr (C.K.); 2 Department of Life Sciences, School of Life and Health Sciences, University of Nicosia, 2417 Nicosia, Cyprus; giannaki.c@unic.ac.cy

**Keywords:** energy drinks, adolescents, sleep debt, obesity, cognition, physical activity, sedentary behavior, caffeine, 24 h movement behavior, public health

## Abstract

Background/Objectives: Energy drinks are marketed to adolescents as products that enhance alertness, concentration, and physical or sports performance, yet their use may also represent a visible marker of sleep debt and a potential contributor to disrupted 24 h behavioral patterns. This narrative review examines evidence linking energy drink consumption with adolescent sleep disturbance and considers implications for obesity risk, cognition, and physical activity. Methods: Targeted literature searches were conducted in PubMed/MEDLINE, ScienceDirect, Google Scholar, and reference lists to identify reviews, consensus statements, policy documents, and adolescent-relevant observational or experimental studies. Evidence was synthesized narratively and classified as strong, moderate, limited, or hypothesis-generating according to study design, consistency, age specificity, and directness to the proposed pathway. Results: The strongest evidence supports associations between energy drink use and short or poor-quality sleep, and between sleep dimensions and adolescent adiposity. Experimental adolescent studies support links between sleep restriction, food choice, reward processing, cognition, and self-regulation. Evidence linking energy drink use directly to reduced physical activity is weaker and likely depends on timing, context, and user subgroup. Sugary and sugar-free products require separate interpretation: sugar-containing drinks may contribute directly to energy intake, whereas sugar-free products may still disrupt sleep through caffeine and may not remove behavioral risk. Conclusions: Energy drink use should be considered not only as caffeine or sugar exposure, but also as a screening cue for sleep debt, fatigue, sedentary behavior, and physical activity readiness. Reducing energy drink use alone is unlikely to resolve adolescent sleep debt; multifactorial interventions that combine caffeine-timing guidance, sleep hygiene, screen management, physical activity support, and policy-level access strategies are needed.

## 1. Introduction

Energy drinks are non-alcoholic beverages containing caffeine and other bioactive ingredients, commonly including taurine, guarana, ginseng, B vitamins, sugars, or non-nutritive sweeteners. They are marketed for alertness, energy, concentration, gaming, studying, sport participation, and social identity [1,2,3]. Consumption among adolescents is widespread. In European data collected for the European Food Safety Authority, 68% of adolescents aged 10–18 years reported having consumed energy drinks at least once during the previous year [2]. Global systematic review evidence also indicates substantial use among adolescents and young adults [4,5,6].

Public health discussion has often focused on caffeine toxicity, sugar intake, cardiovascular symptoms, dental health, marketing, and risk-taking behaviors [1,3,4,5]. These concerns are important, but they may obscure a more everyday pathway: energy drink use may signal, reinforce, or worsen adolescent sleep debt. Adolescents may consume energy drinks because they are already tired, but caffeine exposure, especially later in the day, can delay sleep and increase next-day fatigue. This creates a plausible feedback loop in which fatigue promotes stimulant consumption, stimulant consumption worsens sleep, and sleep debt promotes further fatigue.

This review argues that energy drink use should be considered within sleep medicine, obesity prevention, cognitive health, and 24 h movement behavior. The central claim is not that energy drinks alone cause obesity, cognitive decline, or physical inactivity. Rather, energy drink use may act as a modifiable exposure and marker within a broader phenotype characterized by short or mistimed sleep, daytime sleepiness, unhealthy dietary clustering, low physical activity readiness, sedentary behavior, and increased adiposity. The review also distinguishes stronger evidence from weaker or hypothesis-generating mechanisms, because responsible interpretation is essential in a field dominated by cross-sectional data.

## 2. Methods: Scope, Search Strategy, and Evidence Classification

This article is a focused narrative review and conceptual synthesis. It was not designed as a systematic review, and findings were not pooled quantitatively. The purpose was to integrate evidence across the adjacent literature and propose a testable model linking energy drink use, sleep debt, obesity risk, cognition, and physical activity in adolescents.

Targeted searches were conducted in PubMed/MEDLINE, ScienceDirect, Google Scholar, and reference lists between August 2025 and August 2026. Search terms were combined using Boolean operators and included: energy drink*, caffeinated beverage*, caffeine, adolescent*, youth, sleep duration, sleep quality, sleep debt, insomnia, circadian, obesity, adiposity, body composition, cognition, executive function, academic performance, physical activity, sedentary behavior, fitness, 24 h movement, growth hormone, insulin-like growth factor 1, school policy, and sales restriction. Searches prioritized publications from 2010 onward, while older foundational papers and consensus statements were included when they established key concepts or recommendations.

Eligible sources included systematic reviews, meta-analyses, consensus statements, policy documents, and observational or experimental studies relevant to adolescents aged approximately 10–19 years. Studies including children, adolescents, and young adults were considered when adolescent-specific evidence was limited or when the mechanism was directly relevant to adolescents. Exclusion criteria were: studies focused only on adults without relevance to adolescent physiology or behavior; studies focused on alcohol-mixed energy drinks unless findings were relevant to caffeine or sleep; case reports used as primary evidence for common behavioral outcomes; and papers whose outcomes did not address sleep, obesity, cognition, physical activity, sedentary behavior, caffeine exposure, or policy.

Evidence strength was classified narratively. Evidence was considered strong when supported by systematic reviews or meta-analyses, consistent adolescent observational evidence, or well-established mechanisms directly relevant to the pathway. Evidence was considered moderate when supported by consistent observational findings or adolescent experimental studies but with limitations in generalizability, temporality, or measurement. Evidence was considered limited when adolescent-specific studies were sparse, indirect, inconsistent, or highly context-dependent. Evidence was considered hypothesis-generating when mechanisms were biologically plausible but not directly tested in adolescents or not directly linked to energy drink use. This classification was applied to avoid overstating causal inference and to distinguish energy drink-specific evidence from general caffeine or sleep evidence.

## 3. Energy Drinks as Sleep-Health Exposures

Energy drinks differ from ordinary soft drinks because they are sold as functional stimulant products. The stimulant dose may be difficult for adolescents and parents to estimate, especially when products are sold in large containers, consumed repeatedly, or combined with coffee, cola, pre-workout products, or caffeine-containing medications. Qualitative research suggests that adolescents may view energy drinks as ordinary consumer products rather than pharmacologically active beverages [7].

Overview and systematic review evidence indicates that energy drink consumption in children and young people is associated with short sleep duration, poor sleep quality, psychological distress, risk-taking behavior, substance use, and lower academic performance [4,5]. Adolescent population studies in Australia, Serbia, Korea, Norway, and other settings report associations between regular energy drink consumption and short sleep, late bedtime, low sleep satisfaction, unhealthy dietary clustering, or mental health indicators [8,9,10,11]. These studies cannot prove directionality, but they support the interpretation that energy drink use is embedded within a broader lifestyle pattern.

The clinical question is therefore not only how much caffeine an adolescent consumes, but why, when, and in what behavioral context the product is used. Energy drink consumption during morning sports training, after school, during evening studying, or late-night gaming may carry different implications. The same product may represent performance culture in one adolescent and compensation for chronic sleep debt in another.

## 4. From Energy Drinks to Sleep Debt

Caffeine is an adenosine receptor antagonist. By blocking adenosine-mediated sleep pressure, it can temporarily reduce sleepiness while delaying sleep onset and altering sleep architecture. A systematic review and meta-analysis found that caffeine exposure reduced total sleep time and sleep efficiency, increased sleep onset latency and wake after sleep onset, and reduced deep sleep [12]. Although many experimental caffeine studies are adult studies, the mechanism is relevant to adolescents because delayed circadian timing, early school schedules, and bedtime autonomy already increase vulnerability to insufficient sleep [13,14,15,16].

Adolescent-specific caffeine and energy drink evidence is consistent with this concern. A systematic review of caffeine and adolescent sleep found that most included studies reported shorter total sleep time among caffeine users, although findings for bedtime and sleep onset latency were more variable [17]. Population-based data from Portugal showed that caffeine intake was associated with shorter sleep duration in 13-year-olds [18]. Studies of adolescents in Belgrade, Korea, and Norway also link energy drink use with insufficient sleep, later bedtime, shorter sleep duration, or low sleep satisfaction [9,10,11].

Reverse causality is a major limitation. Adolescents who sleep poorly may be more likely to seek energy drinks the next day. Yet this does not weaken the clinical relevance; it may strengthen it. A bidirectional relationship would mean that energy drink use is both a marker and a potential contributor to sleep debt. In practice, energy drink use should prompt assessment of total sleep opportunity, sleep timing, weekday–weekend variability, daytime sleepiness, evening screen use, and reasons for stimulant use.

## 5. Product Type and Timing: Sugary, Sugar-Free, and Evening Use

A key revision to the model is that energy drink type and timing must be separated. Sugar-containing energy drinks may contribute to obesity risk through direct caloric intake, co-consumption with snacks, and wider clustering with sugar-sweetened beverages and energy-dense foods [8,19]. In contrast, sugar-free energy drinks remove the direct sugar load but do not remove caffeine, behavioral reinforcement, acidic dental exposure, or the possibility of delayed sleep. Therefore, sugar-free products should not be assumed to be sleep-neutral or behaviorally neutral.

Direct adolescent experimental comparisons of sugary versus sugar-free energy drinks on sleep and metabolic outcomes are limited. This gap should be acknowledged explicitly. Available evidence is stronger for caffeine’s sleep-disrupting effects than for product-level comparisons between sweetened and unsweetened energy drinks. Nevertheless, adolescent metabolic evidence raises caution: in a randomized crossover study, caffeine-containing energy shots acutely impaired glucoregulation in healthy adolescents, despite being administered in a controlled experimental context [20]. This finding does not prove long-term obesity effects, but it illustrates that caffeine-containing formulations may have metabolic consequences independent of sugar content.

Timing may be more important than sugar content for sleep. Caffeine taken even several hours before bedtime can impair sleep in experimental studies [21]. A zero-sugar energy drink consumed in the evening may therefore be relevant to sleep debt, next-day fatigue, cognition, and physical activity readiness. Conversely, a sugar-containing product consumed earlier in the day may contribute more directly to energy intake and dietary clustering. Future studies should therefore measure product type, caffeine dose, container size, timing, co-use with other caffeine sources, and consumption context.

## 6. Developmental Stage, Puberty, and Caffeine Vulnerability

Adolescence is not a homogeneous developmental period. Early, middle, and late adolescence differ in pubertal status, circadian timing, autonomy, school demands, body size, risk-taking, and caffeine exposure. Pubertal development is associated with a delay in sleep timing and changes in circadian and homeostatic sleep regulation [14,15,16]. These biological changes occur alongside increasing academic pressure, evening screen use, social autonomy, and reduced parental control of bedtime.

Caffeine vulnerability may therefore differ by developmental stage. Younger or smaller adolescents may receive a higher caffeine dose per kilogram of body mass from the same product than older adolescents. Middle adolescence may be particularly vulnerable because circadian delay, school demands, peer influence, and evening media use often converge. Late adolescents may have more autonomy over purchases, driving, work, sports training, and bedtime, potentially increasing exposure opportunities.

The current evidence base rarely stratifies energy drink-sleep associations by pubertal stage. Most studies use age groups, school grade, or broad adolescent samples. This limits conclusions about developmental windows of risk. Future studies should collect pubertal status, chronotype, sex and gender, body mass, and caffeine dose per kilogram because the same beverage may have different sleep and behavioral implications across developmental subgroups.

## 7. Sleep Debt, Obesity Risk, and Dietary Behavior

Sleep is increasingly recognized as part of obesity prevention in children and adolescents. Prospective meta-analytic evidence indicates that short sleep duration is associated with later obesity risk [22]. An adolescent-focused systematic review further emphasized that multiple sleep dimensions matter: poor sleep hygiene, later chronotype, variable or late sleep timing, and sleep disturbance were consistently associated with greater adiposity [23]. These findings suggest that adolescent adiposity may be linked not only to how long an adolescent sleeps but also to when and how regularly sleep occurs.

Experimental adolescent studies provide plausible dietary mechanisms. Restricted sleep has been associated with greater intake of high-glycemic foods, particularly sweets and desserts [24], greater appeal of sweet/dessert foods [25], and increased carbohydrate and glycemic load intake during late evening eating occasions [26]. These studies do not focus specifically on energy drinks, but they support the pathway from sleep debt to dietary vulnerability. Energy drinks may amplify this pathway when consumed during late-night studying, gaming, screen use, snacking, or socializing.

It is important to avoid overstatement. Energy drink reduction alone has not been shown to prevent adolescent obesity. The defensible claim is that energy drink use may be an upstream marker and possible contributor within a sleep-diet-activity cluster. Asking about energy drinks may reveal actionable information about sleep opportunity, fatigue, late eating, screen exposure, dietary quality, and readiness to engage in movement.

## 8. Cognition, Self-Regulation, and the Stimulant Paradox

Adolescents often consume energy drinks for cognitive reasons: to stay awake, study longer, sustain attention, or improve school performance. The paradox is that repeated stimulant use may preserve wakefulness temporarily while worsening the sleep needed for learning, memory consolidation, emotional regulation, and executive function [27,28].

A randomized crossover trial found that adolescents with higher adiposity were more vulnerable to the negative cognitive effects of one night of sleep restriction on global cognition, fluid cognition, cognitive flexibility, and processing speed than adolescents with lower adiposity [29]. This finding is relevant to obesity prevention because it suggests that sleep debt may not be cognitively neutral in adolescents already at metabolic risk.

Cognition also matters for health behavior. Executive functions, inhibitory control, planning, and emotional regulation influence food choices, screen time, physical activity participation, and adherence to sleep routines [30,31]. A tired adolescent may be less able to plan meals, resist snacks, initiate exercise, or persist in sport. Energy drink use can therefore be interpreted as a signal that the adolescent is attempting to compensate for insufficient sleep through stimulation rather than recovery.

## 9. Physical Activity Readiness: Active and Sedentary Adolescents

The physical activity component of the proposed pathway is plausible but less directly established than the sleep and adiposity links. Physical activity and sleep are bidirectionally related, and the association varies according to timing, intensity, measurement method, and individual characteristics [32,33]. The 24 h movement framework is useful because it treats sleep, sedentary time, and physical activity as co-dependent behaviors across the day rather than isolated exposures [34,35,36].

Energy drinks may be used by physically active adolescents before sport or exercise, by sedentary adolescents to cope with fatigue, or by academically pressured adolescents to extend studying. These scenarios differ. In physically active adolescents, the concern is normalization of stimulant products around sport and possible late-day use after training or competition. In sedentary adolescents, the concern is substitution of stimulation for recovery, potentially reinforcing low energy, screen time, and inactivity. In academically pressured adolescents, the concern is delayed sleep and impaired next-day learning or self-regulation.

The timing of consumption is therefore central. A pre-exercise energy drink consumed early in the day may not have the same sleep consequences as an energy drink consumed in the evening. Conversely, a product used after school, during late gaming, or during night-time studying may be more likely to delay sleep and reduce next-day readiness for physical activity. Future studies should distinguish active sport users, sedentary/fatigued users, and academic/gaming users, rather than treating adolescent energy drink consumers as a single group.

Readiness for physical activity is not identical to measured physical activity. It includes perceived energy, sleepiness, mood, soreness, motivation, self-efficacy, and confidence to participate. If adolescents arrive at school or practice sleep restricted, energy drinks may temporarily mask sleepiness but not restore recovery. This is a hypothesis requiring direct testing with objective sleep, accelerometry, sedentary time, fitness, caffeine timing, and repeated measures of fatigue and motivation.

## 10. Endocrine and Anabolic Recovery: Plausible but Limited Evidence

The endocrine and anabolic aspects of the pathway should be framed cautiously. Growth hormone secretion is linked to slow-wave sleep, and sleep supports growth, tissue repair, musculoskeletal adaptation, immune function, and recovery from physical activity [37]. However, it is not established that energy drink consumption directly reduces growth hormone secretion in adolescents. Acute sleep disruption may also not uniformly suppress pulsatile growth hormone secretion in pubertal children [38].

For this reason, growth hormone and insulin-like growth factor 1 should not be presented as the primary causal mechanism. They are better viewed as part of a broader recovery framework. The strongest statement is that chronic sleep debt may reduce the adolescent’s opportunity for nocturnal biological and behavioral restoration. This includes endocrine rhythms, but also attention, mood, appetite regulation, glucose metabolism, and physical recovery.

## 11. Confounding Variables and Effect Modifiers

The proposed feedback loop is likely modified by several contextual factors. Socioeconomic status may influence access to energy drinks, food environments, school schedules, safe spaces for activity, parental monitoring, and sleep opportunity. Screen time is another major confounder because it can delay sleep, increase sedentary behavior, expose adolescents to marketing, and co-occur with snacking and energy drink consumption. Chronotype, school start time, homework load, employment, sport schedules, and social stress may also shape the relationship.

Neurodiversity should be considered explicitly. Adolescents with attention-deficit/hyperactivity disorder may have different baseline sleep problems, stimulant medication exposure, executive-function challenges, and patterns of caffeine use. Studies suggest that caffeine use and sleep problems may be clinically relevant in adolescents with ADHD [39]. The implication is not that energy drink use has uniform effects across neurodevelopmental groups, but that caffeine and sleep should be assessed carefully where ADHD, learning difficulties, or mental health symptoms are present.

These confounders do not invalidate the model. Instead, they identify where the model should be tested and where interventions should be individualized. A causal pathway from energy drinks to sleep debt, inactivity, and obesity cannot be isolated without measuring these contextual variables.

## 12. Proposed 24 h Behavioral Model

**Figure 1:**
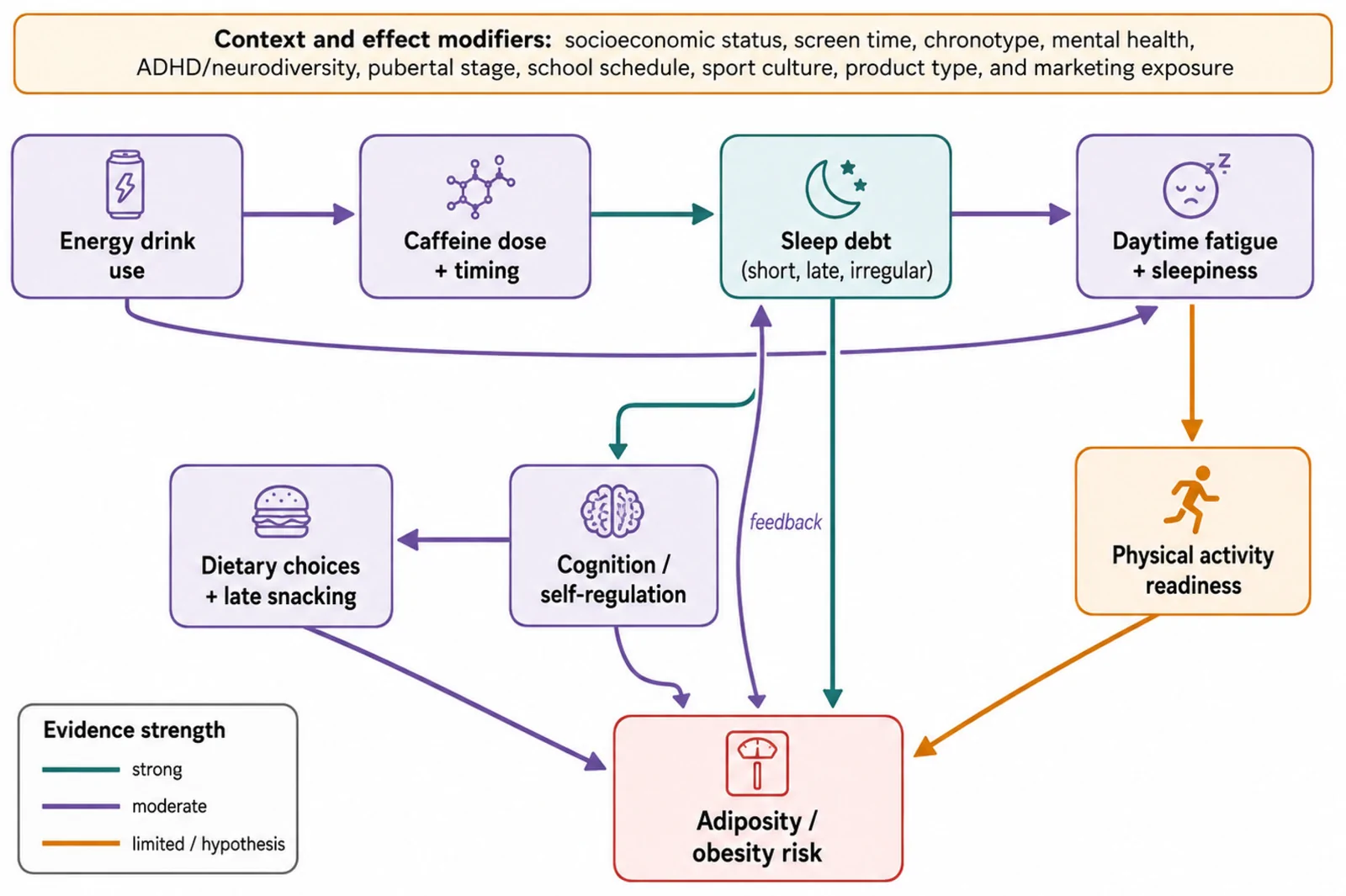
Proposed 24 h behavioral model linking adolescent energy drink use, sleep debt, cognition, physical activity readiness, and obesity risk. Line thickness and color indicate relative evidence strength. The model is intended as a testable framework, not proof of a single causal chain.

Figure 1 presents a conceptual model. The model is a feedback system rather than a simple linear chain. Energy drink use may originate from sleep debt, academic pressure, sport culture, gaming, peer norms, taste preference, or marketing. Once consumed, especially later in the day, caffeine may delay or fragment sleep. Reduced sleep can worsen daytime fatigue, cognitive control, mood, appetite regulation, and food reward. These changes can increase the likelihood of further energy drink use, higher intake of sweet or energy-dense foods, sedentary behavior, and lower readiness for physical activity. Increased adiposity may then worsen sleep through psychosocial, respiratory, inflammatory, or behavioral pathways, reinforcing the loop.

The strongest links in the model are energy drink or caffeine exposure with sleep disruption, and sleep dimensions with adiposity. Evidence for sleep restriction affecting food choice and cognition is moderate and supported by adolescent experimental studies. Evidence linking energy drink use specifically to reduced physical activity is limited and context-dependent. The model therefore does not claim that energy drinks directly cause adolescent obesity through inactivity. It proposes that energy drink use may be a modifiable entry point into a broader sleep debt and 24 h behavior phenotype.

## 13. Clinical Screening and Tiered Assessment

For clinicians, energy drink use should be treated as a screening cue. A two-level process may improve practicality (Table 1). Initial screening should be brief and feasible in pediatric primary care, obesity clinics, sleep clinics, sports medicine, and school health services. It should ask how often energy drinks are used, at what time they are consumed, how much caffeine is likely present, whether other caffeine sources are used, whether the adolescent uses them because of tiredness, and whether sleep duration is below the recommended 8–10 h per 24 h [13].

In-depth assessment should follow when the adolescent presents with obesity, insomnia, daytime sleepiness, school problems, attention difficulties, or low participation in physical activity. For adolescents with obesity, the focus should include late eating, appetite, sleep timing, snoring or sleep-disordered breathing symptoms, sedentary time, and readiness for movement. For adolescents with sleep complaints, assessment should emphasize caffeine timing, weekday-weekend sleep variability, evening screen exposure, and insomnia symptoms. For adolescents with learning difficulties, attention problems, or ADHD, the focus should include self-medication motives, stimulant medication timing, sleepiness, and executive function challenges. For active adolescents, questions should distinguish sport-related use from evening compensatory use.

Counseling should avoid a simplistic message that abstinence alone will solve sleep debt. Avoiding afternoon and evening caffeine is practical, but it may not be sufficient if the adolescent’s sleep opportunity remains too short, screens remain active late at night, or academic and social demands compress bedtime. Energy drink assessment should lead to problem-solving around sleep opportunity, caffeine timing, screen routines, physical activity support, and family or school environment.

## 14. School and Public Health Priorities

Interventions should be prioritized according to feasibility, scope, and evidence. Short-term school actions include removing energy drinks from school sales and vending, educating students and parents about caffeine timing, integrating sleep education into health curricula, and training school staff to recognize energy drink use as a possible marker of fatigue. These actions are feasible and can be evaluated using school-level consumption, sleepiness, attendance, and classroom functioning indicators.

Medium-term clinical and community strategies include routine screening for energy drink use, sleep timing, daytime sleepiness, sedentary behavior, and physical activity readiness in pediatric, obesity, sleep, and sports medicine settings. Community programs should combine sleep hygiene, screen management, accessible physical activity, and healthy beverage alternatives rather than relying solely on warnings.

Long-term policy options include age-of-sale restrictions, marketing restrictions, clearer caffeine labeling, limits on package size or price promotions, and restrictions on product placement around schools or youth sport. A 2024 scoping review identified diverse policy strategies to reduce energy drink consumption among children, including taxation, labeling, sales restrictions, school bans, and marketing regulation [40]. In England, the Department of Health and Social Care has confirmed a plan to introduce legislation banning sales of high-caffeine energy drinks to children under 16, defining in-scope drinks as those, other than tea or coffee, containing more than 150 mg of caffeine per liter [41].

Policy should not rely on prohibition alone. Reviewer concern on this point is well taken: prohibitionist policies and information-only campaigns may be insufficient if adolescents use energy drinks because their environments restrict sleep. Regulatory approaches should therefore be paired with sleep-health promotion, screen-time interventions, family education, school schedule considerations, and evaluation of unintended substitutions, such as increased coffee, caffeine tablets, or pre-workout product use.

## 15. Limitations and Research Agenda

The proposed pathway requires stronger evidence. First, many studies rely on self-reported energy drink use and sleep. Future studies should collect product-level data, caffeine dose, container size, sugar content, non-nutritive sweetener status, time of consumption, co-use with coffee or cola, and context such as studying, gaming, sport, or socializing. Second, sleep should be measured with actigraphy or other objective methods where feasible, with attention to duration, timing, variability, sleep onset latency, wake after sleep onset, and daytime sleepiness. Third, physical activity and sedentary behavior should be assessed continuously across several days rather than by broad questionnaire categories. Fourth, prospective and experimental designs are needed. Cross-sectional associations cannot determine whether energy drinks cause poor sleep, whether poor sleepers seek energy drinks, or whether both reflect third variables such as screen time, socioeconomic stress, ADHD, chronotype, or sensation seeking. Randomized trials that reduce afternoon and evening caffeine exposure, combined with sleep extension or sleep hygiene support, could test whether sleep, daytime fatigue, dietary choices, cognitive function, and movement behavior improve.

Fifth, outcomes should extend beyond body mass index. Body composition, waist circumference, cardiorespiratory fitness, muscular fitness, insulin sensitivity, mood, cognitive function, school attendance, injury risk, and quality of life are relevant. Sex, gender, pubertal stage, neurodevelopmental status, and socioeconomic context should be evaluated as moderators. Mechanistic studies may include growth hormone or insulin-like growth factor 1, but these markers should be interpreted within broader recovery, metabolic, cognitive, and behavioral outcomes rather than as isolated biomarkers.

## 16. Conclusions

Energy drink use in adolescence should be taken seriously as a sleep-health exposure and as a possible marker of insufficient recovery. The current evidence supports a cautious but actionable position: energy drink use should trigger assessment of sleep timing, daytime sleepiness, diet quality, sedentary behavior, and physical activity readiness. The evidence is strongest for energy drinks and poor sleep, and for sleep dimensions and adiposity; it is more limited for direct effects on physical activity and anabolic endocrine pathways.

The goal is not simply to remove a beverage. Reducing energy drink exposure is most likely to be useful when paired with adequate sleep opportunity, screen management, healthy dietary environments, and support for enjoyable physical activity. If future studies confirm the proposed pathway, energy drink assessment may become a practical entry point into adolescent obesity prevention, cognitive-health promotion, and 24 h lifestyle medicine.

## 17. Practice Points

- Energy drink use in adolescents should be considered a sleep-health exposure and a potential marker of sleep debt, not only a caffeine or sugar issue.
- Sugar-containing and sugar-free energy drinks require different interpretations: sugar-containing products add calories, while sugar-free products may still disrupt sleep through caffeine and reinforce stimulant-use behavior.
- The strongest evidence supports associations between energy drink consumption and short or poor-quality sleep, and between sleep disturbance and adolescent adiposity.
- Evidence linking energy drink use directly to reduced physical activity is currently limited and should be framed as a context-dependent hypothesis involving timing, fatigue, motivation, and recovery.
- Clinical assessment should include energy drink timing, reasons for use, sleep opportunity, daytime sleepiness, screen time, sedentary behavior, and physical activity readiness.
- Public health strategies should combine school-level actions, clinical screening, sleep hygiene, screen management, and policy measures rather than relying on prohibition or information alone.

## 18. Research Agenda

- Prospective studies should test whether energy drink use predicts later changes in sleep timing, sleep duration, physical activity, cognition, and adiposity.
- Experimental studies should evaluate whether reducing afternoon and evening caffeine exposure improves sleep, daytime fatigue, food choice, cognitive function, and movement behavior.
- Studies should measure product type, caffeine dose, sugar content, non-nutritive sweetener status, container size, timing of consumption, co-use with other caffeine sources, and context of use.
- Objective measurement of sleep, sedentary time, and physical activity should be prioritized using actigraphy or accelerometry where feasible.
- Analyses should stratify or adjust for pubertal stage, sex and gender, chronotype, socioeconomic status, screen time, ADHD/neurodiversity, and sport participation.
- Mechanistic studies may include growth hormone or insulin-like growth factor 1, but these markers should be interpreted within broader recovery, metabolic, cognitive, and behavioral outcomes.

## Figures and Tables

**Table 1:** Tiered clinical assessment of adolescent energy drink use within sleep, obesity, cognitive, and physical activity contexts.

Clinical Context	Initial Screening	In-Depth Assessment	Action Focus
All adolescents	Frequency, timing, product size, reason for use, other caffeine sources	Sleep duration, weekday-weekend variability, daytime sleepiness, evening screens	Caffeine timing guidance and sleep opportunity
Obesity or adiposity concern	Energy drink type, sugar content, late eating, fatigue	Snoring, sleep-disordered breathing symptoms, appetite, sedentary time, movement barriers	Integrate sleep and beverage counseling with obesity care
Sleep complaint	Evening caffeine, insomnia symptoms, wake time, naps	Actigraphy or sleep diary where feasible; screen exposure; chronotype	Reduce late caffeine and improve sleep routines
Learning, attention, or ADHD concerns	Use for studying, concentration, or self-medication; medication timing	Sleepiness, executive function, anxiety, stimulant co-use	Coordinate sleep, mental health, and school support
Sport or high activity	Pre-exercise vs. evening use; product confusion with sports drinks	Training time, recovery, injury, sleep after late practice	Separate sport hydration from stimulant products

## Data Availability

No new data were created or analyzed in this narrative review. Data sharing is not applicable to this article.

## References

[1] Schneider M.B., Benjamin H.J., Committee on Nutrition and the Council on Sports Medicine and Fitness (2011). Sports drinks and energy drinks for children and adolescents: Are they appropriate?. Pediatrics.

[2] Zucconi S., Volpato C., Adinolfi F., Gandini E., Gentile E., Loi A., Fioriti L. (2013). Gathering consumption data on specific consumer groups of energy drinks. EFSA Support. Publ..

[3] Curran C.P., Marczinski C.A. (2017). Taurine, caffeine, and energy drinks: Reviewing the risks to the adolescent brain. Birth Defects Res..

[4] Ajibo C., Van Griethuysen A., Visram S., Lake A.A. (2024). Consumption of energy drinks by children and young people: A systematic review examining evidence of physical effects and consumer attitudes. Public Health.

[5] Khouja C., Kneale D., Brunton G., Raine G., Stansfield C., Sowden A., Sutcliffe K., Thomas J. (2022). Consumption and effects of caffeinated energy drinks in young people: An overview of systematic reviews and secondary analysis of UK data to inform policy. BMJ Open.

[6] Aonso-Diego G., Krotter A., Garcia-Perez A. (2024). Prevalence of energy drink consumption world-wide: A systematic review and meta-analysis. Addiction.

[7] Visram S., Crossley S.J., Cheetham M., Lake A.A. (2017). Children and young people’s perceptions of energy drinks: A qualitative study. PLoS ONE.

[8] Nuss T., Morley B., Scully M., Wakefield M. (2021). Energy drink consumption among Australian adolescents associated with a cluster of unhealthy dietary behaviours and short sleep duration. Nutr. J..

[9] Tomanic M., Paunovic K., Lackovic M., Djurdjevic K., Nestorovic M., Jakovljevic A., Markovic M. (2022). Energy drinks and sleep among adolescents. Nutrients.

[10] Kim B., Lee S.G., Kim T.H. (2023). Poor sleep is associated with energy drinks consumption among Korean adolescents. Public Health Nutr..

[11] Kaldenbach S., Leonhardt M., Lien L., Bjaertnes A.A., Strand T.A., Holten-Andersen M.N. (2022). Sleep and energy drink consumption among Norwegian adolescents: A cross-sectional study. BMC Public Health.

[12] Gardiner C., Weakley J., Burke L.M., Roach G.D., Sargent C., Maniar N., Townshend A., Halson S.L. (2023). The effect of caffeine on subsequent sleep: A systematic review and meta-analysis. Sleep Med. Rev..

[13] Paruthi S., Brooks L.J., D’Ambrosio C., Hall W.A., Kotagal S., Lloyd R.M., Malow B.A., Maski K., Nichols C., Quan S.F. (2016). Recommended amount of sleep for pediatric populations: A consensus statement of the American Academy of Sleep Medicine. J. Clin. Sleep Med..

[14] Crowley S.J., Wolfson A.R., Tarokh L., Carskadon M.A. (2018). An update on adolescent sleep: New evidence informing the perfect storm model. J. Adolesc..

[15] Carskadon M.A. (2011). Sleep in adolescents: The perfect storm. Pediatr. Clin. N. Am..

[16] American Academy of Pediatrics (2014). School start times for adolescents. Pediatrics.

[17] Bonnar D., Gradisar M. (2015). Caffeine and sleep in adolescents: A systematic review. J. Caffeine Res..

[18] Lodato F., Araujo J., Barros H., Lopes C., Agodi A., Barchitta M., Ramos E. (2013). Caffeine intake reduces sleep duration in adolescents. Nutr. Res..

[19] Malik V.S., Hu F.B. (2022). The role of sugar-sweetened beverages in the global epidemics of obesity and chronic diseases. Nat. Rev. Endocrinol..

[20] Shkembi B., Huppertz T. (2020). Caffeine-containing energy shots cause acute impaired glucoregulation in adolescents. Nutrients.

[21] Drake C., Roehrs T., Shambroom J., Roth T. (2013). Caffeine effects on sleep taken 0, 3, or 6 hours before going to bed. J. Clin. Sleep Med..

[22] Miller M.A., Kruisbrink M., Wallace J., Ji C., Cappuccio F.P. (2018). Sleep duration and incidence of obesity in infants, children, and adolescents: A systematic review and meta-analysis of prospective studies. Sleep.

[23] Gale E.L., Williams A.J., Cecil J.E. (2024). The relationship between multiple sleep dimensions and obesity in adolescents: A systematic review. Sleep Med. Rev..

[24] Beebe D.W., Simon S., Summer S., Hemmer S., Strotman D., Dolan L.M. (2013). Dietary intake following experimentally restricted sleep in adolescents. Sleep.

[25] Simon S.L., Field J., Miller L.E., DiFrancesco M., Beebe D.W. (2015). Sweet/dessert foods are more appealing to adolescents after sleep restriction. PLoS ONE.

[26] Duraccio K.M., Whitacre C., Krietsch K.N., Zhang N., Summer S., Price M., E Saelens B., Beebe D.W. (2022). Losing sleep by staying up late leads adolescents to consume more carbohydrates and a higher glycemic load. Sleep.

[27] Tarokh L., Saletin J.M., Carskadon M.A. (2016). Sleep in adolescence: Physiology, cognition and mental health. Neurosci. Biobehav. Rev..

[28] de Bruin E.J., van Run C., Staaks J., Meijer A.M. (2017). Effects of sleep manipulation on cognitive functioning of adolescents: A systematic review. Sleep Med. Rev..

[29] Stager L.M., Watson C.S., Cook E.W., Fobian A.D. (2024). Effect of sleep restriction on adolescent cognition by adiposity: A randomized crossover trial. JAMA Neurol..

[30] Nelson T.D., Nelson J.M., Mason W.A., Tomaso C.C., Kozikowski C.B., Espy K.A. (2019). Executive control and adolescent health: Toward a conceptual framework. Adolesc. Res. Rev..

[31] Beebe D.W. (2011). Cognitive, behavioral, and functional consequences of inadequate sleep in children and adolescents. Pediatr. Clin. N. Am..

[32] Lang C., Kalak N., Brand S., Holsboer-Trachsler E., Puehse U., Gerber M. (2016). The relationship between physical activity and sleep from mid adolescence to early adulthood: A systematic review of methodological approaches and meta-analysis. Sleep Med. Rev..

[33] Master L., Nye R.T., Lee S., Nahmod N.G., Mariani S., Hale L., Buxton O.M. (2019). Bidirectional, daily temporal associations between sleep and physical activity in adolescents. Sci. Rep..

[34] Tremblay M.S., Carson V., Chaput J.P., Connor Gorber S., Dinh T., Duggan M., Faulkner G., Gray C.E., Gruber R., Janson K. (2016). Canadian 24-Hour Movement Guidelines for Children and Youth: An integration of physical activity, sedentary behaviour, and sleep. Appl. Physiol. Nutr. Metab..

[35] Bull F.C., Al-Ansari S.S., Biddle S., Borodulin K., Buman M.P., Cardon G., Carty C., Chaput J.-P., Chastin S., Chou R. (2020). World Health Organization 2020 guidelines on physical activity and sedentary behaviour. Br. J. Sports Med..

[36] Wilhite K., Booker B., Huang B.H., Antczak D., Corbett L., Parker P., Noetel M., Rissel C., Lonsdale C., Cruz B.d.P. (2023). Combinations of physical activity, sedentary behavior, and sleep duration and their associations with physical, psychological, and educational outcomes in children and adolescents: A systematic review. Am. J. Epidemiol..

[37] Zaffanello M., Pietrobelli A., Cavarzere P., Guzzo A., Antoniazzi F. (2024). Complex relationship between growth hormone and sleep in children: Insights, discrepancies, and implications. Front. Endocrinol..

[38] Calvert M.E., Molsberry S.A., Kangarloo T., Amin M.R., Genty V., Faghih R.T., Klerman E.B., Shaw N.D. (2022). Acute sleep disruption does not diminish pulsatile growth hormone secretion in pubertal children. J. Endocr. Soc..

[39] Cusick C.N., Langberg J.M., Breaux R., Green C.D., Becker S.P. (2020). Caffeine use and associations with sleep in adolescents with and without ADHD. J. Pediatr. Psychol..

[40] Rostami M., Babashahi M., Ramezani S., Dastgerdizad H. (2024). A scoping review of policies related to reducing energy drink consumption in children. BMC Public Health.

[41] Department of Health and Social Care (2026). Banning the Sale of High-Caffeine Energy Drinks to Children: Consultation Outcome.

